# Optimized convolutional neural network using African vulture optimization algorithm for the detection of exons

**DOI:** 10.1038/s41598-025-86672-x

**Published:** 2025-01-30

**Authors:** K. Jayasree, Malaya Kumar Hota

**Affiliations:** Department of Communication Engineering, School of Electronics Engineering, Vellore Institute of Technology, Vellore, Tamil Nadu 632014 India

**Keywords:** Convolutional neural network (CNN), Exons, Modified Gabor wavelet transform (MGWT), Three base periodicity properties (TBP), African vulture optimization algorithm (AVOA), Computational biology and bioinformatics, Energy science and technology

## Abstract

The detection of exons is an important area of research in genomic sequence analysis. Many signal-processing methods have been established successfully for detecting the exons based on their periodicity property. However, some improvement is still required to increase the identification accuracy of exons. So, an efficient computational model is needed. Therefore, for the first time, we are introducing an optimized convolutional neural network (optCNN) for classifying the exons and introns. The study aims to identify the best CNN model that provides improved accuracy for the classification of exons by utilizing the optimization algorithm. In this case, an African Vulture Optimization Algorithm (AVOA) is used for optimizing the layered architecture of the CNN model along with its hyperparameters. The CNN model generated with AVOA yielded a success rate of 97.95% for the GENSCAN training set and 95.39% for the HMR195 dataset. The proposed approach is compared with the state-of-the-art methods using AUC, F1-score, Recall, and Precision. The results reveal that the proposed model is reliable and denotes an inventive method due to the ability to automatically create the CNN model for the classification of exons and introns.

## Introduction

In bioinformatics, the growth of genomic signal processing (GSP) has drastically increased in the last two decades for identifying protein-coding regions. In eukaryotic DNA, detection of the protein-coding region in the gene is a challenging task because short protein-coding regions (exons) are interrupted by the long non-coding regions (introns)^1^. The coding regions are the conserved part of genomes for identifying and transferring biological genetic information during protein synthesis^2^. Each protein has a specific three-dimensional structure based on the sequence of amino acids in the coding regions. The structure and function of proteins can be changed by a genetic mutation, which leads a diseases like cancer and genetic disorders. Therefore, accurate identification of protein-coding regions is required to understand the structure of the protein which is further helped in drug design and diagnosis of genetic diseases^3^.

The GSP is used to analyze DNA sequences based on the signal processing approaches for coding region identification by utilizing the exon’s essential three-base periodicity (TBP) property^4^. The TBP property occurs due to the non-uniform usage of codons (groups of three adjacent nucleotides), also known as codon bias: even though several codons could code a given amino acid, they are not used with uniform probability in organisms^11^.

Several methods have been proposed for identifying the protein-coding region based on DSP techniques. The character strings in the DNA sequences are converted into numerical sequences before the application of DSP methods. Initially, Tiwari et al.^5^ proposed the short-time discrete Fourier transform (ST-DFT) to differentiate coding and non-coding regions. To improve the performance of ST-DFT, Anastassiou et al.^6^ proposed enhanced frequency-domain visualization tools, and Kotlar et al.^7^ employed spectral rotation characteristics. These methods use a rectangular window with a fixed window length. This fixed window length is not suitable for all the DNA sequences and also the rectangular window causes the spectral leakage. To overcome these, an adaptive window length approach was proposed by Shakya et al.^8^. To reduce these a fuzzy adaptive window median filter^9^ and an adaptive Kaiser window^10^ methods have been used.

To reduce the spectral leakage problem, other signal processing methods based on digital filters have been developed. Vaidyanathan and Yoon^11^ introduced an anti-notch filter having a central frequency of *f*/3 for exon detection. Several filtering approaches were established to improve identification accuracy by reducing background noise^12–16^. Nevertheless, the filter’s specific parameters might not apply to various DNA datasets. Recently fine-tuned variational mode decomposition based on kurtosis and ST-DFT has been developed for better coding region identification^17^.

The constraint of the ST-DFT method is its dependence on window characteristics such as shape and length^18^. These problems were resolved by using multiresolution transform methods such as modified Gabor wavelet transform (MGWT)^19^, wide-range wavelet window^20^, fuzzy adaptive Gabor wavelet transform^21^, and MGWT with signal boosting technique^22^.

Many researchers used DSP methods to detect the exons in eukaryotic DNA sequences and achieved good accuracy. In the last few decades, machine learning^23^ and deep learning (DL)^24^ algorithms have become more popular for identifying and classifying signals in many fields. The DL algorithm is more sophisticated due to its ability to expand the depth of the neural network’s internal layers^24^. One of the deep neural network (DNN) technologies used recently is the convolutional neural network (CNN). Because of its high classification accuracy and prediction, many researchers motivated and applied it to various applications^25–33^. The structure of the CNN model consists of many layers including convolutional, ReLu, pooling, and fully connected layers, and is designed to automatically learn the special hierarchies of features by extracting the significant features in its initial layers and complex features in deeper layers. Every layer has its corresponding hyperparameters such as the number of filters and the kernel size of each convolutional layer, the kernel size of the pooling layer, the number of hidden units in the fully connected layer, and so on. The depth of CNN, which refers to the number of convolutional, pooling, and fully connected layers with its hyperparameters, as well as the training options like optimizer and the batch size plays a major role in accurate prediction and classification. Therefore, the design of the CNN model with its parameters for the particular dataset is a challenging task. If the depth of the CNN increases then its corresponding hyperparameters also increase. Typically, choosing these hyper-parameters is done manually through an expensive trial-and-error process. However, the overall efficiency of the CNN model depends on the appropriate hyperparameters as well as the depth of the CNN. Hence the construction of the CNN model and the selection of its hyperparameters are considered as an optimization problem. A metaheuristic is a more sophisticated technique that is intended to locate, produce, adjust, or choose a strategy that might offer a suitable response to an optimization or an artificial intelligence problem. Recently, many researchers implemented metaheuristic algorithms for tuning the parameters in various applications^24,25,34–38^.

As per the literature, the characteristics of the signal are identified based on the learning process of the deep learning algorithm, and the accuracy is increased using the optimization methods.

This motivates us to classify exons and introns in the eukaryotic DNA sequences by employing the appropriate CNN model. To improve the accuracy, the structure of the CNN model and its hyperparameters are optimized using the African vulture optimization algorithm (AVOA). The AVOA algorithm has gained the attention of researchers and it has been used in various fields to solve optimization problems due to its simplicity, fast convergence rate, flexibility, and effectiveness^39^.

The main contribution of the paper is summarized as follows:

1. A novel computational approach is proposed that can automatically construct and identify an appropriate layered architecture with satisfactory performance.
2. This research work optimizes the hyperparameters of the layered architecture of the CNN model using the African Vulture Optimization Algorithm (AVOA).
3. The AVOA algorithm is adapted for CNN parameter optimization. An interpretation is used to convert the population created for the functioning of the AVOA to the population of particles whose information is comprehensible by the CNN.
4. The best-layered architecture with its hyperparameters generated by the AVOA, enables us to classify the exons and introns efficiently.

## Preliminaries

### Dataset

In this work, two benchmark datasets such as the GENSCAN training set^40^ and the HMR195^41^ are considered for extracting coding regions in eukaryotic DNA to evaluate the proposed method. The GENSCAN training set consists of 380 genes, in that 238 are multi-exon genes and 142 are the single exon gene sequences of humans. The HMR195 dataset has 195 genes, in that 43 are single-exon genes and 152 are multiple-exon gene sequences of human, mouse, and rat sequences in the ratio of 103:82:10. In this dataset, the proportion of coding and non-coding sequences is 14% and 86% respectively. Further, the average number of exons per gene is 4.86. Therefore, it is very challenging to identify the coding regions appropriately.

### MGWT

The performance of the ST-DFT depends on the window length. The predetermined window length reduces identification accuracy. This limitation is overcome by employing multi-scale analysis techniques on DNA sequences containing both large and small protein-coding regions. Mena-Chalco et al.^19^. proposed a modified Gabor-wavelet transform (MGWT) that can analyze the existence of a particular frequency (or periodicity) in a DNA sequence at a regularly varying scale.

The mutation of the Gabor-wavelet function for evaluating a DNA sequence in a particular frequency and multiple scales is defined as

1 $$\:{\phi\:}_{MGWT}\left(x,a,b\right)={e}^{\frac{{(x-a)}^{2}}{2{b}^{2}}}{e}^{j{\omega\:}_{0}\left(x-a\right)}$$

where $\:a$ is the position, $\:b>0$ is the scale parameter and the center frequency of $\:{\phi\:}_{MGWT}$ is represented as $\:{\omega\:}_{0}$.

Hence, the MGWT is expressed as a function of $\:a$ and$\:b$ as

2 $$\:U\left(a,b\right)=\int\:u\left(x\right){e}^{\frac{{(x-a)}^{2}}{2{b}^{2}}}{e}^{j{\omega\:}_{0}\left(x-a\right)}$$

The spectrum of every sequence is described as the complex squared modulus of their MGWT coefficients and represented by

3 $$\:{w}_{\beta\:}\left(a,b\right)=\sum\:{\left|{U}_{\beta\:}(a,b)\right|}^{2}$$

where ∈ {*A*, *C*, *G*, *T*}. To explore the TBP components, the entire spectrum of indicator sequences is calculated for different scales ‘$\:b$ ' at a particular frequency $\:{\omega\:}_{0}=N/3$.

### Convolutional neural network

In recent years, CNN has worked as an important area for artificial intelligence (AI) research. However, due to its high performance in learning and generalizing various problems like classification, identification, and segmentation, it is widely used in many fields including engineering, medicine, and the defence sector. In general, the CNN structure consists of three basic layers: The Convolutional layer, the Pooling layer, and the Fully connected layer.

### Convolutional layer

This layer is the fundamental structural component of CNN. The structure of this layer is made up of several filters called kernels and it generates feature maps after performing the convolution between the input layer and the filters. To identify several features, the number of convolutional layers increases accordingly.

### Pooling layer

This layer is used to decrease the dimensions of the feature while retaining the input properties. Thus, the computational complexity is reduced. In 1-D CNN, maximum and average pooling layers are the standard types of pooling. The goal of this layer is to preserve the low-frequency components of the signal while eliminating its high-frequency components.

### Fully connected layer

This is the most essential layer of CNN architecture used for classification. It trains the network to perform the learning process by considering the data from the previous layer.

According to the CNN principle, the relevant data received by the input layer is transferred through the convolutional and pooling layers and then passes to the last fully connected layer. During the network training, the error obtained between the desired data and the output of the fully connected layer is reduced by updating the weights using an optimization algorithm. This training process is continued until it reaches the desired epoch value.

### African vulture optimization algorithm (AVOA)

The AVOA is a new metaheuristic algorithm that draws motivation from the environment, proposed by Abdollahzadeh et al.^42^. The AVOA simulates the searching behavior of African vultures. The vultures are divided into three categories based on their physical characteristics: the strongest vultures belong to the first group, the weaker vultures belong to the second group, and the weakest vultures belong to the third group. In this algorithm, the population of possible vultures is initialized randomly, and this first group of candidates is comparable to the vultures that started their food search. The method used to determine how many vultures are needed for a specific task is also employed to determine the population. Because of its adaptability, the algorithm can adjust its performance to fit a variety of optimization challenges. The African vulture algorithm follows four stages: choosing the best vulture from any population grouping, vulture hunger rates, exploration, and exploitation.

## Methodology to implement AVOA for optimizing the structure and its hyperparameters of CNN

**Fig. 1:**
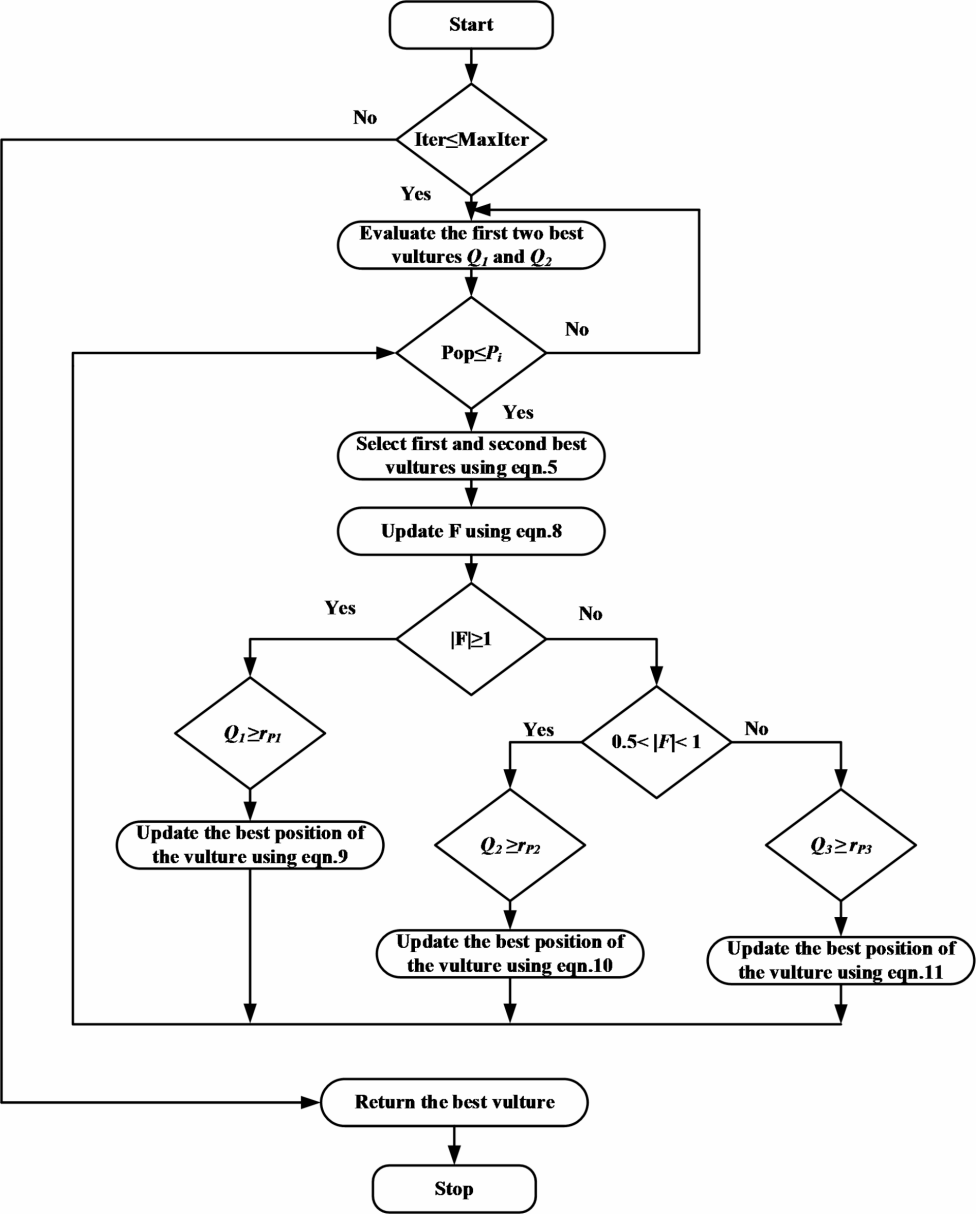
Flow chart of AVOA.

This session explains the steps to implement AVOA for optimizing the structure and parameters of the CNN model with the flow chart shown in Fig. 1. Vultures denote the candidate indicating the number of CNN model layers and hyperparameters (solutions).

*Step 1.* Set the parameters of the AVOA and maximum iterations Imax. Initialize the random population of vultures N and the solution vectors R(i). Load the HMR195 dataset and the GENSCAN training set.

*Step 2.* Evaluate the fitness function for every solution for the present iteration using the following equation 

4 $$\:Bit\:error\:rate\:\left(BER\right)=\frac{FP+FN}{TP+FP+TN+FN}$$

*Step 3.* Determine the values of the first and second-best fitness functions as the first-best 1 and second-best 2 solutions in two different groups.

*Step 4.* Determine *X(j)* from Eq. (5) using the roulette wheel criterion, then choose one of the two best solutions from step 3 to be the current best for the present iteration.

5 $$\:X\left(j\right)=\left\{\begin{array}{c}Best\_Vultre1\:ifqi=L1\\\:Best\_Vultre2\:\:if\:qi=L2\end{array}\right.$$

6 $${\text{and}}\,{q}_{i}=\frac{{F}_{j}}{\sum\:_{j=1}^{n}{F}_{j}}$$

where, $\:{F}_{j}\to\:{j}_{th}$ vulture fitness value. $\:L1$ and $\:L2$ are the random numbers in the range [0, 1]. X(j) is one of the fitness values.

*Step 5.* Find the vulture satisfaction F, using Eqs. (7 and 8), which determines whether the vulture is searching in the exploration or exploitation mode.

7 $$\:u=h*\left({sin}^{w}\left(\frac{\pi\:}{2}*\frac{Iter\_j}{{Max}_{j}}\right)+cos\left(\frac{\pi\:}{2}*\frac{Iter\_j}{{Max}_{j}}\right)-1\right)$$

8 $$\:F=\left(2*{rand}_{1}+1\right)*z1*\left(1-\frac{Iter\_j}{{Max}_{j}}\right)+u$$

where w is the fixed numerical, z1 is the random number range [-1,1], $\:Iter\_j$ and $\:{Max}_{j}$ are current and maximum iterations, respectively.

*Step 6.* Begin exploration: If *|F|* > 1 verify the conditions on parameter *Q*_*1*_ and update the current-best using Eq. (9) if not go to step 7.

9 $$\:Q\left(j+1\right)=\left\{\begin{array}{c}\left(X\left(j\right)-\left(\left|T*X\left(j\right)-Q\left(j\right)\right|\right)*F\right)\:if\:{Q}_{1}\ge\:{r}_{p1}\\\:X\left(j\right)-F+{r}_{2}*\:\left(\left(ub-lb\right)*{r}_{3}+lb\right)\:if\:{Q}_{1\:}<{r}_{p1}\end{array}\right.$$

T is a coefficient vector, $\:Q\left(j+1\right)$ is the vulture position vector in the next iteration, $\:ub$ and $\:lb$ are the upper and lower bounds of the variables, $\:{r}_{3}$ and $\:{r}_{p1}$are the random numbers ranges between [0,1].

*Step 7.* Begin exploitation: If 0.5< *|F|*< 1 verify the parameters *Q*_*2*_ and update the current-best using Eq. (10) else verify the parameters *Q*_*3*_ and update *Q(j)* by using Eq. (11).

10 $$\:Q\left(j+1\right)=\left\{\begin{array}{c}\left(\left|T*X\left(j\right)-Q\left(j\right)\right|\right)*\left(F+{r}_{4}\right)-\left(X\left(j\right)-Q\left(j\right)\right)\\\:if\:{Q}_{2}\ge\:{r}_{p2}\\\:X\left(j\right)-X\left(j\right)*\left(\frac{Q\left(j\right)}{2\pi\:}\right)\left[{r}_{5\:}*cos\:\left(\:Q\left(j\right)\right)+{r}_{6\:}*sin\left(\:Q\left(j\right)\right)\right]\\\:if{Q}_{2}<{r}_{p2}\end{array}\right.$$

where, *r*_p2_,* r*_4_, *r*_5_, and *r*_6_ are the random numbers in the range [0, 1].

11 $$\:Q\left(j+1\right)=\left\{\begin{array}{c}\frac{1}{2}\left[{BV}_{1}\left(j\right)+{BV}_{2}\left(j\right)-\left(\frac{{BV}_{1}\left(j\right)*Q\left(j\right)}{{BV}_{1}\left(j\right)-{Q\left(j\right)}^{2}}+\frac{{BV}_{2}\left(j\right)*Q\left(j\right)}{{BV}_{2}\left(i\right)-{Q\left(i\right)}^{2}}\right)*F\right]\\\:\begin{array}{c}if{Q}_{3}\ge\:{r}_{P3}\\\:X\left(j\right)-\left(\left|X\left(j\right)-Q\left(i\right)\right|\right)*F*LF\left(d\right)\\\:if\:{Q}_{3}<{r}_{P3}\end{array}\end{array}\right.$$

were *r*_*P3*_ is the random numbers in the range [0 1], $\:{BV}_{1}\text{t}\text{h}\text{e}$ best vulture of 1st group, $\:{BV}_{2}$best vulture of 2nd group, *LF* is the Levy Flight function, and *d* is the problem dimensions.

*Step 8.* If population ≤ Max population go to step 4, else verify the stopping condition Imax, and if it satisfies, save the current-best as best-result else go to step 2.

## The proposed CNN

**Fig. 2:**
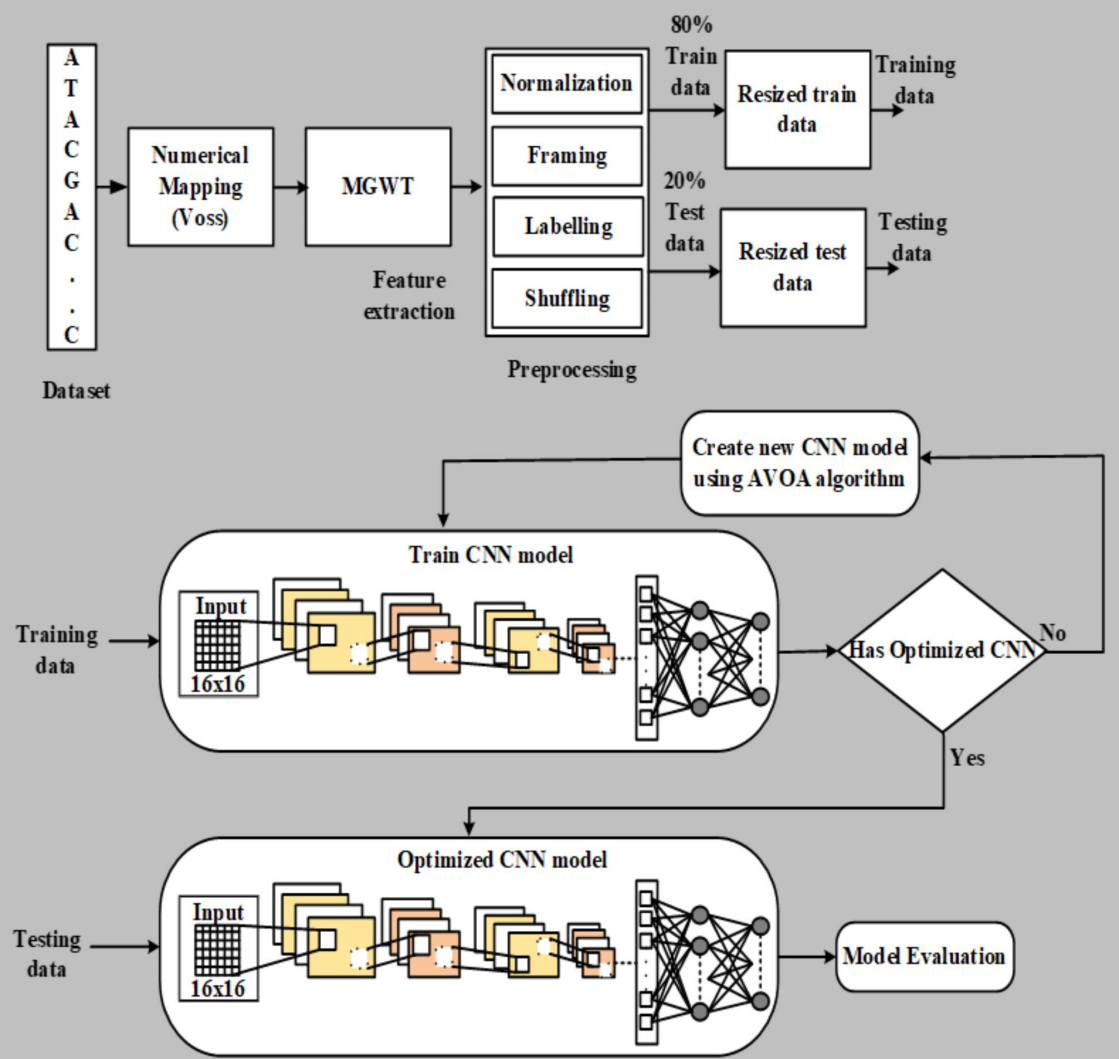
The architecture of the proposed AVOA-based optCNN model.

The architecture of the proposed AVOA-based Optimized CNN model (AVOA-optCNN) is illustrated in Fig. 2 for detecting the exons in the eukaryotic DNA sequence.

The AVOA-optCNN is a hybrid model concentrated on fetching the advantages of each of the involved algorithms. The main reason for selecting the CNN model in this work is due to its high performance in learning and generalizing the classification problems. On the other hand, the AVOA is a metaheuristic algorithm, which has demonstrated reliability in identifying global solutions within the feasible space. It has efficient balancing among the exploitation and exploration stages so that it will efficiently satisfy the objective function.

The proposed method has three main sections such as (1) numerical mapping (2) extracting appropriate features and (3) optimizing the structure and hyperparameters of the CNN using the AVOA algorithm and training the optimized CNN using the resultant optimized hyperparameters.

### Numerical mapping

A DNA sequence is made up of four nucleotide base pairs of adenine (*A*), cytosine (*C*), guanine (*G*), and thymine (*T*). These DNA sequences with symbol strings cannot be directly analysed using DSP-based techniques. Thus, the string form of the DNA sequence is converted into numerical form using some numerical mapping approaches. Although several mapping techniques have been presented, selecting a proper mapping method is important for extracting the TBP features.

For numerical conversion, we have employed Voss mapping^43^ methods in this work. the Voss mapping technique is frequently utilized by researchers, because of its excellent performance in GSP^5,19–21,44,45^. This fixed binary mapping technique converts the DNA sequences into four binary indicator sequences that indicate the presence (binary ‘1’) and absence (binary ‘0’) of each nucleotide.

### Feature extraction

In the next step, it was necessary to extract the feature from the signals using an efficient method for the proper classification of exons and introns. In this work, the MGWT approach is used to extract the TBP components of the coding region from the dataset and the obtained DNA spectrum is considered as the feature. After extracting the features, some preprocessing steps are necessary to make them suitable for processing and training the CNN model. First, the features are normalized into the range of [0 1]. Then the normalized features are separated into two classes according to their periodicity. Later the periodic and non-periodic signals are divided into multiple frames, each with a length of 256 and an overlapping of 100 samples. The data needs to be prepared for the CNN by converting the vector form of each data into a 16*16 matrix. After that, a class is allocated to every frame according to its periodicity. The frame is categorized as class 1 if it has periodic features; if not, it is considered as class 2.

All these frames are randomly shuffled to ensure that every class is distributed evenly throughout each set. Then this data is split into 80% for training purposes and the remaining 20% for testing. The detailed description of the datasets used in the simulation is illustrated in Table 1.

**Table 1:** Details of training and testing datasets for CNN.

Name of the dataset	Total number of signals	Total number of classes	Number of signals in training set (80%)	Number of signals in the testing set (20%)
HMR195	8885	2	7108	1777
GENSCAN Training set	14,164	2	11,331	2833

### The proposed optimization of CNN structure using AVOA

As can be seen from the previous explanation, the CNN model consists of different layers, each layer has specific characteristics. Although CNNs performed extremely well in solving many classification issues, choosing the right CNN structure for a particular application is challenging. Therefore, the AVOA algorithm in the proposed method is used to obtain the best CNN model that will provide the maximum accuracy for the classification of exons and introns. The AVOA searches for the particles that enable the CNN to get the appropriate results in the classification problems.

This global search is accomplished by minimizing the fitness function (*BER*) represented in Eq. (1). The AVOA is responsible for selecting the best architecture of the CNN to achieve acceptable performance.

The first stage of the AVOA-optCNN process is the initialization of a random population of N candidates, each of which is defined in a three-dimensional space. These dimensions represent the number of convolutional, pooling, and fully connected (FC) layers respectively. For each type of layer, the related hyperparameters are assigned. The CNN can comprehend the layered architecture when each candidate performs the transformation procedure of the initial population.

The CNN starts its training by using the training DNA sequences from the dataset and the configuration of each candidate that creates the entire population. The neural network determines the corresponding *BER* for each particle and stores this value as a local best (*P*_*j*_^*best*^), which is utilized by the AVOA during its optimization process. This procedure is repeated until the final candidate from the initial population is completed.

The AVOA determines the best vulture (V_gbest_) by using the candidate j whose value *P*_*j*_^*best*^ is the smallest of all the BER values predicted for the complete initial population at the end of this iterative cycle. At each iteration, AVOA aims to minimize the value of BER and updates the position of each candidate by considering the position and V_gbest_ value.

The search for the V_gbest_ is repeated until it reaches the maximum iteration value. The V_gbest_ is the global best candidate in the population that gives satisfactory solutions while training the CNN model for the classification of exons and introns. Once the modifications are finished, the layer arrangement denoted by the V_gbest_ is considered to estimate the performance of the CNN model using test data from the HMR195 dataset and the GENSCAN training set.

**Fig. 3:**
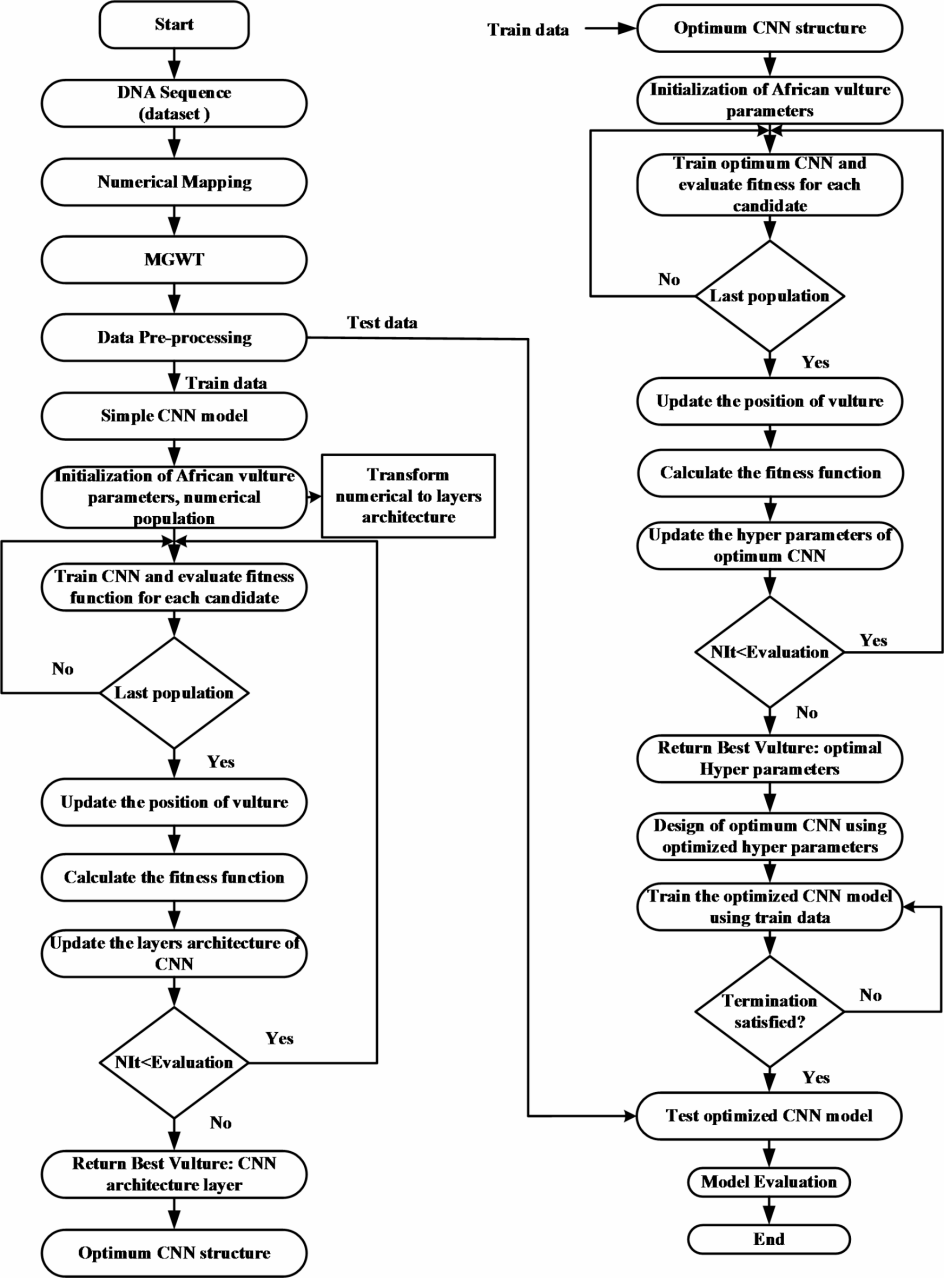
Flow chart of the proposed AVOA-based optCNN.

The flow chart of the AVOA-optCNN is shown in Fig. 3, and the pseudo-code of the proposed method is illustrated in Algorithm 1.

**Algorithm 1:**
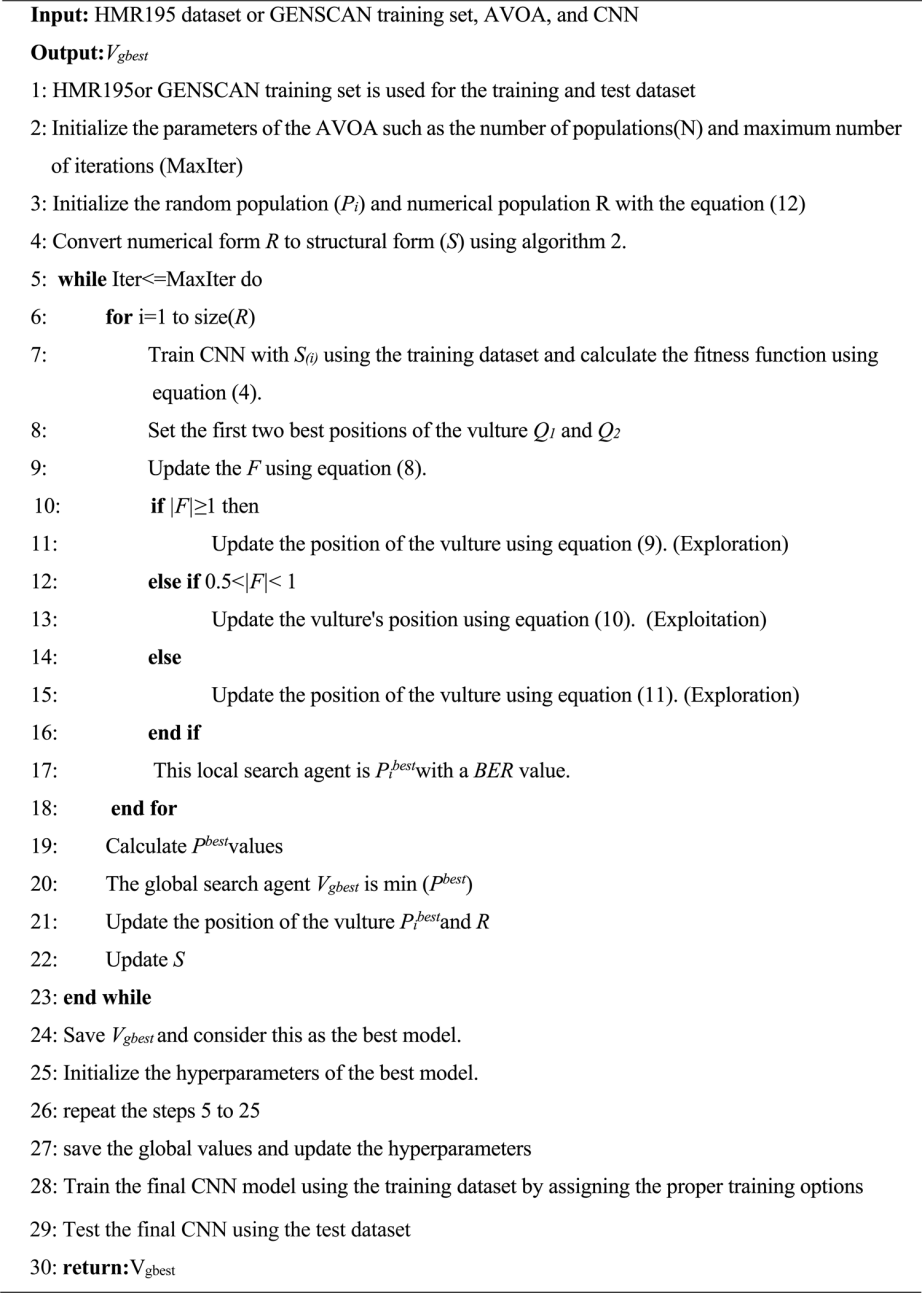
The Proposed AVOA-based CNN method.

The flow chart and pseudo code explain the methodology of the proposed model used in this work. The optimization algorithm AVOA requires the population in numerical form. Therefore, the proposed method offers a conversion strategy between the numerical population (*R*) for the AVOA and the population consisting of several structures of layer architectures (*S*). The CNN requires this conversion to understand and solve the classification problems. While *R* allows the AVOA to perform optimization work, *S* allows the CNN to perform classification tasks and calculate the objective function of the AVOA.

### Initialize the numerical population

In general, meta-heuristic algorithms demand a search space of feasible solutions that are determined by a given population consisting of a specific number of entities. The main goal of the possible solutions is to increase or decrease the value of a fitness function. In the proposed method, the individuals that are made up of candidates of the AVOA algorithm are defined by an R matrix having dimensions of $\:\left(K\times\:L\right)$:

12 $$\:R=\left(\begin{array}{cccc}{S}_{\text{1,1}}&\:{S}_{\text{1,2}}&\:\cdots\:&\:{S}_{1,L}\\\:{S}_{\text{2,1}}&\:{S}_{\text{2,2}}&\:\cdots\:&\:{S}_{2,L}\\\:⋮&\:⋮&\:\ddots\:&\:⋮\\\:{S}_{K,1}&\:{S}_{K,2}&\:\cdots\:&\:{S}_{K,L}\end{array}\right)$$

Assuming that K is the total number of particles in R and L is the number of dimensions that AVOA updates, the dimensions of R collectively form the solution search space. In this case, the L value is 3. These three dimensions have directly correlated with the number of CNN layers. The numerical value specifically corresponds to the number of convolutional layers for L = 1, the number of pooling layers for L = 2, and the number of fully connected layers for K = 3.

Thus, $\:{S}_{K,L}$ represents the values adopted for each dimension that compose the population and its content is determined by the equivalent value of *L*. The content of each candidate *K* depends on the minimum and maximum number of layers.

To perform the AVOA-optCNN, it is essential to consist of at least two layers in the architecture. The first layer is the convolutional layer and the last layer is the fully connected layer having some neurons that must be equal to the number of classes that need to be predicted. The maximum number of layers depends on the complexity of the input. The $\:{S}_{K,1},{S}_{K,2},{S}_{K,3}$ is calculated by using a function randint.

13 $$\:{S}_{K,1}=randint(\text{m}\text{i}\text{n}_\text{C},\:\text{m}\text{a}\text{x}_\text{C})$$

14 $$\:{S}_{K,2}=randint(\text{m}\text{i}\text{n}_\text{p},\:\text{m}\text{a}\text{x}_\text{p})$$

15 $$\:{S}_{K,3}=randint(\text{m}\text{i}\text{n}_\text{F},\:\text{m}\text{a}\text{x}_\text{F})$$

where min_C_, min_p_, min_F_, max_C_, max_p_, and max_F_ are the integers that depend on the complexity of input data and the number of target classes to predict. It is necessary to create an optimal structure of the CNN, the optimization process of AVOA uses the numerical values present in each dimension of matrix *R* by minimizing the objective function of each candidate and it generates different layer architectures in numerical form.

Therefore, converting the *R* population to a data structure that CNN can easily understand is essential in this work.

### Transform numerical population to layered architecture

The conversion of the numerical nature of R to a data structure that enables the functionality of the CNN correctly is required for the classification. The different architectures of each candidate in the *R* matrix are stored in the data structure matrix *Y* is defined as:

16 $$\:Y=\left(\begin{array}{c}{Z}_{\text{1,1}}\\\:{Z}_{\text{2,1}}\\\: \vdots \\\:{Z}_{K,1}\end{array}\right)$$

Thus, for each $\:{Z}_{K,1}$, a different layer architecture is stored. The values present in the $\:{S}_{K,1}$, $\:{S}_{K,2}$, $\:{S}_{K,3}$, is converted to their corresponding $\:{Z}_{K,1}$ by using the Algorithm 2.

**Algorithm 2:**
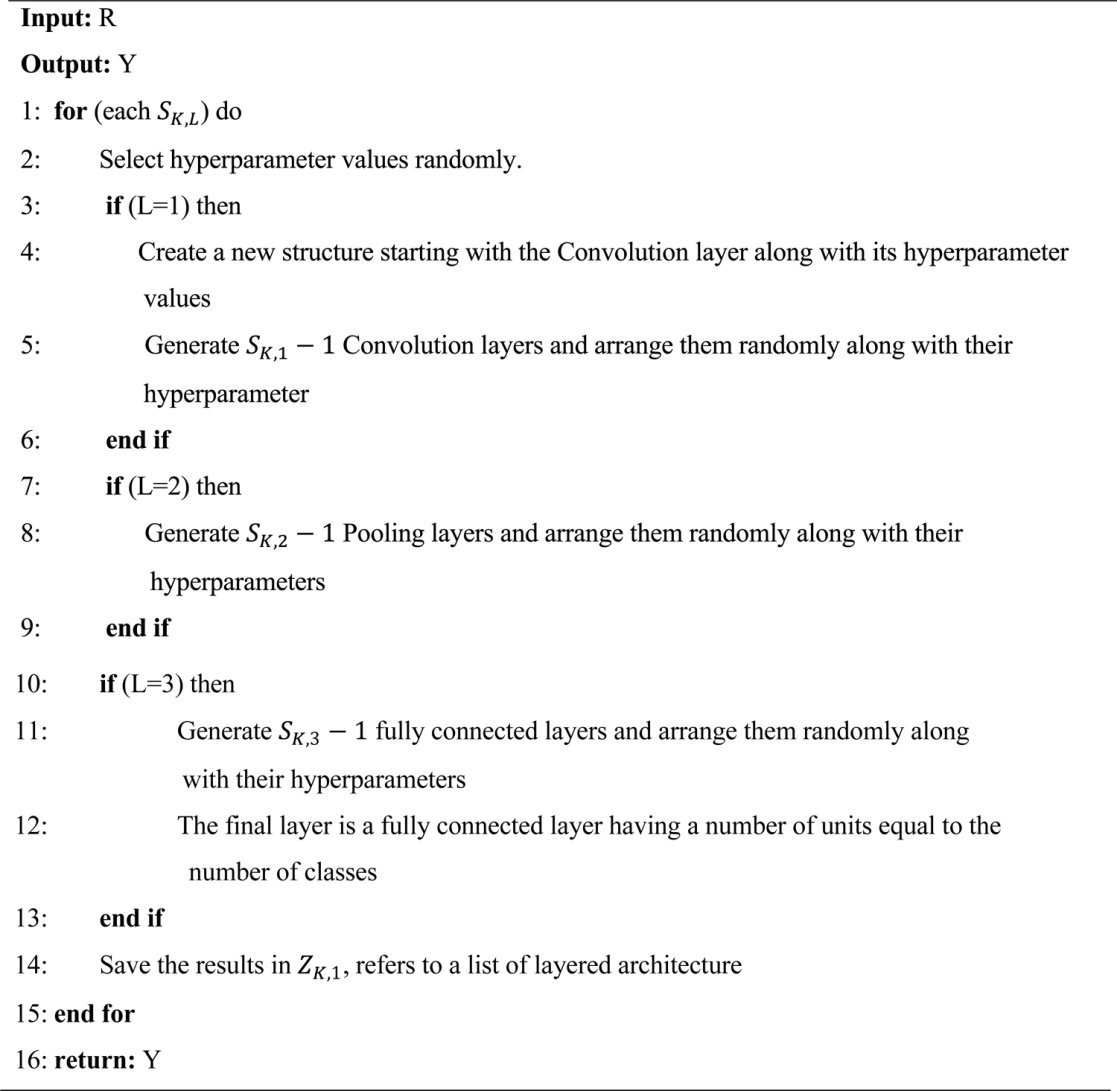
Converting numerical population to layered architecture.

**Fig. 4:**
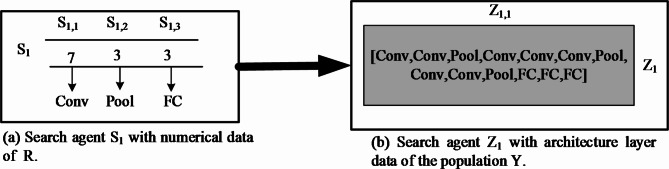
Convert numerical data to architecture layer data.

This algorithm receives the input that the values contain the *R*. The first layer of any candidate is always the convolution layer, and its hyperparameters are randomly selected. The last layer is fully connected, with the neurons equal to the number of classes that need to be classified. The remaining layers are placed in between these layers and the positions of their places are selected randomly. For example, Fig. 4 represents the contents of search agents with a total number of layers is 13, and the data for this search agent corresponds to the population of R. For converting the numerical search agents *S*_*1*_ to a structural form *Z*_1_, algorithm 2 is used. The architecture built for that search agent is stored in *Z*_1,1_, and *S*_1,1_ contains the value corresponding to the number of convolution layers. This type of layer is positioned randomly between the pooling and Conv in the architecture. The value found in *S*_*1*,2_ represents the number of pooling layers and position is given similarly to the Conv. In this work, Max pooling is used as the subtype of pooling. Finally, the position of *S*_1,3_ represents the whole number of fully connected layers and its position indicates the last layer of the architecture *Z*_1,1_.

When the conversion process from R to S is finished, the CNN model evaluates each candidate in S and then begins its training and classification process by calculating the *BER* value.

### Update process

For every candidate of *S*, the optCNN computes a fitness value (*BER*), which is then taken as a *p*_*j*_^*best*^ and its corresponding position is updated. The fitness values and their associated position for each candidate in the whole population enclosed in S are represented as a vector. The AVOA yields the best global candidate (V_gbest_) from this vector by considering the smallest value of all the evaluated *BER*.

Since the updating process is carried out on the *R* matrix, it is essential to update the *Y* data structure using the new values determined by the AVOA, once it is completed estimating the new values and candidate positions.

This process is repeated until the AVOA reaches the total number of iterations. After the final iteration, the proposed CNN stores the layered structure based on the V_gbest_.

## Hyperparameters optimization of CNN model using AVOA

Once the CNN layered structure is defined, the hyperparameters are selected based on the optimization algorithm so that it gives the highest accuracy for solving any classification problems. In this work, the hyperparameters of the selected CNN structure are optimized using AVOA through four steps: parameter selection, population initialization, estimation of the objective function, and updating the position.

In parameter selection, the parameters of the CNN such as the number of filters (N_f_) and filter size (*F*_*s*_) related to the convolutional layer. Kernel size (*P*_*s*_) of the pooling layer and number of hidden units (H) in fully connected layers are represented as a vector having m number of parameters. It is calculated as *m=(2c + p + h)* where *c* is the number of convolutional layers, *p* is the number of pooling layers and *h* is the number of hidden layers.

Once the CNN hyperparameters are selected, the initial population, consisting of n candidates, is randomly initialized. After initialization, the model is trained with the appropriate dataset to find the fitness value of a candidate solution until it converges. Finally, the CNN structure’s optimized hyperparameters are determined by calculating the global best fitness value with the lowest *BER*.

### Classification metrics

The performance of the proposed CNN model is evaluated using quantitative evaluation parameters such as confusion matrix, Precision, Accuracy, F1-score, and Recall.

17 $$\:Precision=\frac{TP}{TP+FP}$$

18 $$\:Recall=\frac{TP}{TP+FN}$$

19 $$\:F1-score=\frac{2TP}{2TP+FP+FN}$$

20 $$\:\text{A}ccuracy=\frac{TP+TN}{TP+TN+FP+FN}$$

## Experimental results

In this section, the effectiveness of the CNN model obtained based on the optimization method is validated by comparing it with some existing methods. The performance of the proposed method depends on the optimization algorithm. Therefore, in this work five optimization algorithms such as the African vulture optimization algorithm (AVOA), Particle swarm optimization algorithm (PSO)^46^, Educational competition optimizer (ECO)^47^, Parrot optimizer (PO)^48^, and Rime optimization algorithm (RIME)^49^ are introduced for creating the optimized layered architecture of the CNN along with its parameters.

**Fig. 5:**
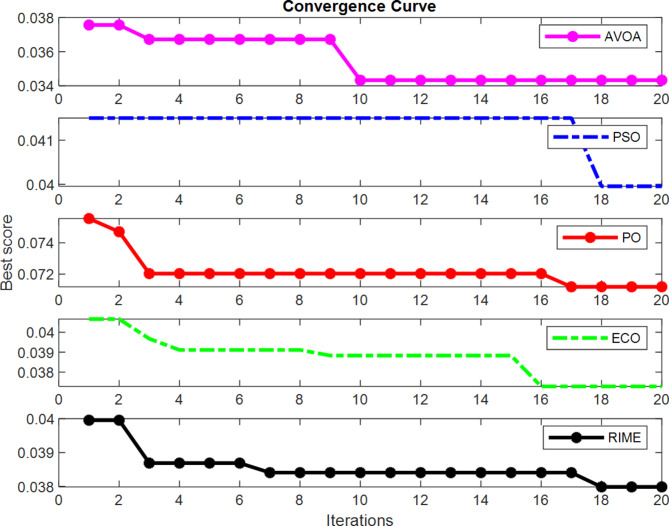
Convergence Curve during minimization of the fitness function.

The convergence curves of the algorithms AVOA, PSO, ECO, PO, and RIME are shown in Fig. 5 for the HMR195 dataset. Figure 5 shows that the AVOA converges faster than the other optimization methods and the fitness value is also minimum than other methods. Therefore, this work uses the AVOA optimization algorithm to design an optimal CNN architecture.

## Experiment setup and parameters used in the proposed method

The initialization parameters used in this work for the AVOA algorithm are shown in Table 2. Here, the number of populations is described as 10 where each population represents a CNN structure. Hence, 10 different structures are formed in each iteration. In this case, the number of iterations is selected as 20. The *Q1*, *Q2*, and *Q3* are the controlling parameters of AVOA, and *L1*, and *L2* are the random numbers selected between 0 and 1.

**Table 2:** Initial parameters for the AVOA.

Name of Parameters	Value
Q1	0.6
Q2	0.4
Q3	0.6
Alpha (L1)	0.8
Betha (L2)	0.2
Gamma (w)	2.5
Maximum iteration	20
Population size	10

The range of hyperparameter values used in this work for the optimization of the CNN model is described in Table 3. While optimizing the structure of the model, the minimum number of layers is set to 3 and the maximum number of layers is set to 11. The minimum required layers used in the CNN architecture are convolutional, pooling, and fully connected. The batch normalization and ReLu layers are placed after the convolutional layer. The fully connected layer is always placed at last in the CNN architecture.

**Table 3:** Parameters values of CNN structure.

Name of Parameter	Range of value
Number of layers	[3–11]
Number of filters in the convolutional layer	[8 16 32]
The size of filters in the convolutional layer	[3 × 3], [5 × 5], [7 × 7]
Size of kernel in pooling layer	[2–5]
Hidden units in a fully connected layer	[10–1024]
Mini batch size	[8 16 32 64 128]
Optimizer	‘adam’, ‘sgdm’, ‘rmsprop’

The layers in the CNN structure consist of hyperparameters such as the number of filters, kernel size of the convolutional layer, the filter size of the pooling layer, and the number of hidden units in the fully connected layer. The kernel size of the convolutional layer is used for selecting the features and the number of filters is used for selecting the features for the next layers. Therefore, the selection of these parameters is essential. If the kernel size is too small then it fails to capture the information of neighbours and if it is too high, which leads to ignoring the fine details. So, in this work, the filer sizes are selected as [3 × 3], [5 × 5], or [7 × 7]. The number of filters is selected as 8,16 or 32. The pooling size is used for controlling the pooling layer which is used for down-sample the feature. In this work, the range for pooling layer size is between [2 × 2] to [5 × 5]. The number of hidden units in the fully connected layers ranges from 10 to 1024.

The parameters used for training the CNN algorithm are illustrated in Table 4. The selection of the mini-batch size is essential at the time of training. Here the mini-batch size is selected in the range [8,16,32, 64 or 128]. During the training of the CNN model, an appropriate optimizer needs to be selected to reduce the error. The number of epochs and the learning rate are fixed at 10 and 0.001 respectively. In this work, the first 10 epochs are used for optimizing the layered architecture, the next 10 epochs are used for optimizing the hyperparameters of the optimum CNN model, and the final 10 epochs are used for training the optimized CNN model.

**Table 4:** Parameters for training the CNN model.

Name of parameter	Range of value
optimizer	‘adam’, ‘sgdm’, ‘rmsprop’
Epoch used to create the optimum CNN model	10
Epoch used for selecting the hyperparameters of the optimum CNN model	10
Epoch used to train the optimized CNN	10
Mini batch size	[8 16 32 64 128]
Initial learning rate	0.001

## Optimized layer architecture of CNN model obtained with adapted AVOA

The best-optimized CNN architectures obtained for the HMR 195 and GENSCAN training datasets are called optCNN-HMR and optCNN-GenTrain respectively. The layered architecture and its hyperparameters of these models are represented in Table 5 (a) and (b) respectively. The optCNN-HMR model consists of 12 layers, including three convolutional layers, one pooling layer, and two fully connected layers. In that, the first layer is always a convolutional layer and the last layer is the fully connected layer having the number of neurons equal to the number of classes. The optCNN-GenTrain model consists of 17 layers, including four convolutional layers, three pooling layers, and two fully connected layers. In this work, the number of classes is two. After every convolutional layer batch normalization and ReLu layers are added.

**Table 5:** The best-layered architecture of the CNN model with optimal hyperparameters using AVOA algorithm (a) for the dataset HMR 195 and (b) for the dataset GENSCAN Training set.

optCNN-HMR			optCNN-GenTrain		
Layer	Parameters	Value	Layer	Parameters	Value
Conv1	No.of filters	8	Conv1	No.of filters	16
	Filter size	3		Filter size	3
Conv2	No.of filters	16	Max pooling layer	Kernel size	3 × 3
	Filter size	5	Conv2	No.of filters	16
Conv3	No.of filters	16		Filter size	5
	Filter size	3	Max pooling layer	Kernel size	2 × 2
Max pooling layer	Kernel size	2 × 2	Conv3	No. of filters	32
Fully connected	No. of hidden units	1024		Filter size	3
	Optimizer	‘sgdm’	Max pooling layer	Kernel size	2 × 2
	Batch size	8	Conv4	No. of filters	32
				Filter size	5
			Fully connected	No. of hidden units	1024
				Optimizer	‘sgdm’
				Batch size	32
(a)			(b)		

## Results of optimized CNN model

**Fig. 6:**
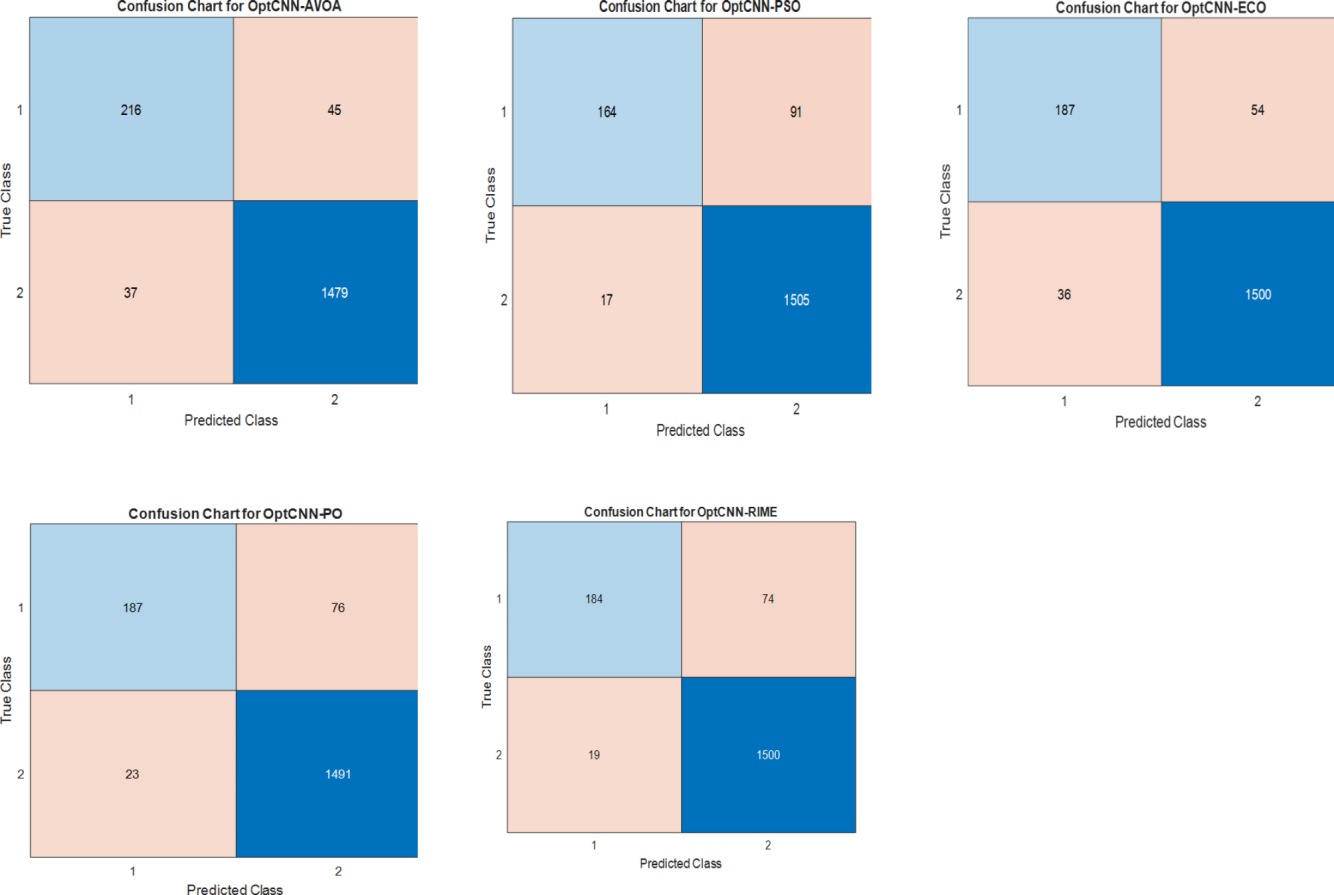
Confusion matrices of the optimized CNN model using the HMR195 dataset.

**Fig. 7:**
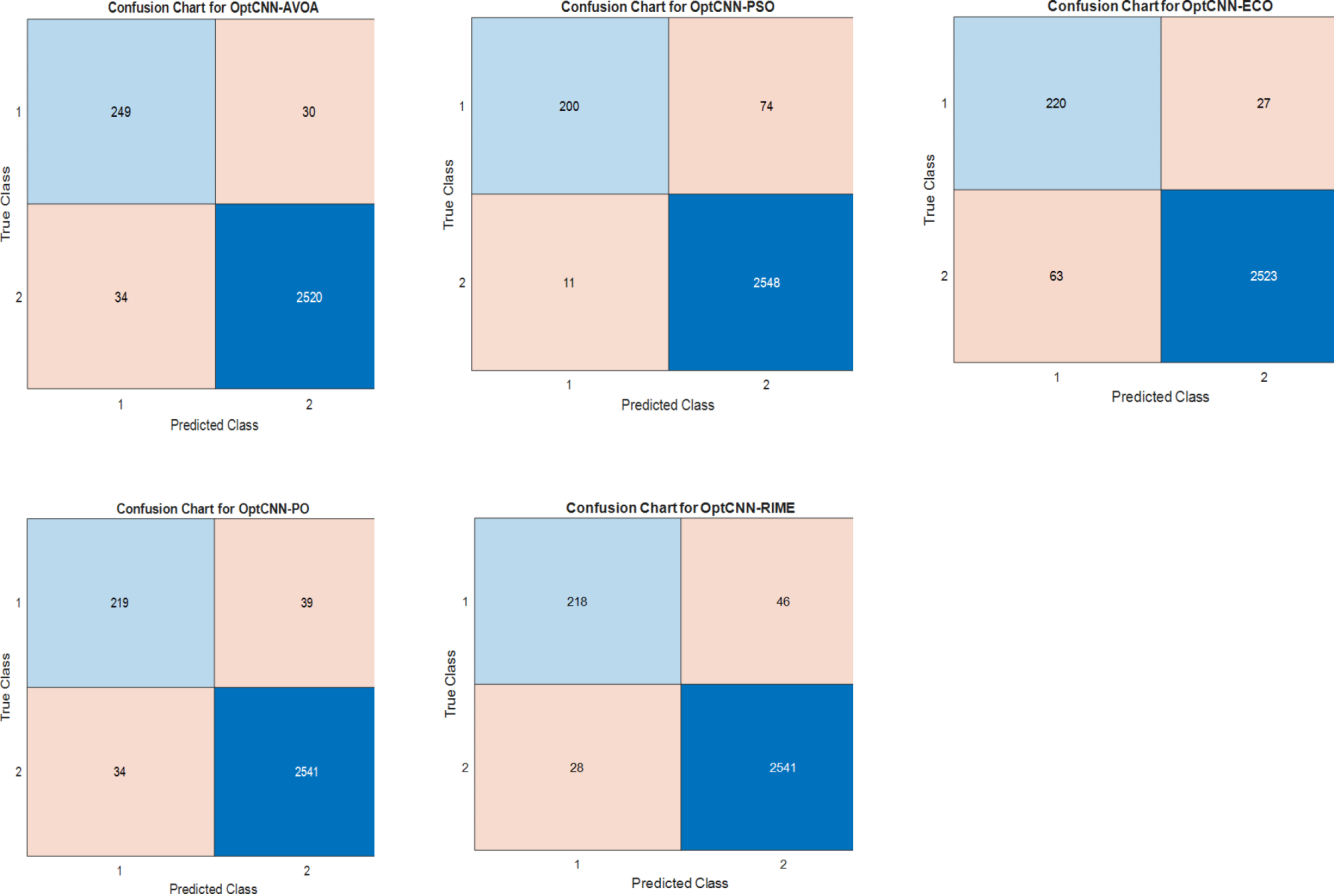
Confusion matrices of the optimized CNN model using the GENSCAN training dataset.

It is crucial in this field to accurately predict the presence of exons in the DNA sequence since these lays a strong foundation for protein synthesis. To validate the performance of the proposed method, different evaluation metrics such as confusion chart, Accuracy, F1-score, Precision, and Recall are used. The confusion metrics compute the true positive (TP), false positive (FP), false negative (FN), and true negative (TN). The confusion matrix of the optCNN-HMR and optCNN-GenTrain models for different optimization algorithms are shown in Figs. 6 and 7. A comparative analysis of the proposed method and other existing approaches for the HMR195 and GENSCAN training datasets are illustrated in Tables 6 and 7.

**Table 6:** Comparison of the proposed and existing methods for the dataset HMR195.

Metrics	ST-DFT	MGWT	RIME-optCNN	PO-optCNN	ECO-optCNN	PSO-optCNN	AVOA-optCNN
Accuracy	0.7512	0.7761	0.9477	0.9443	0.9494	0.9392	0.**9539**
AUC	0.8069	0.8402	0.9751	0.9676	0.9655	0.9624	0.**9801**
F1-score	0.5815	0.5979	0.7983	0.7907	0.8050	0.7523	0.**8405**
Precision	0.4724	0.5270	0.9064	0.8905	0.8386	0.8061	0.**8638**
Recall	0.6810	0.7238	0.7132	0.7110	0.7759	0.6431	0.**8276**

**Table 7:** Comparison of the proposed and existing methods for the dataset GENSCAN training set.

Metrics	ST-DFT	MGWT	RIME-optCNN	PO-optCNN	ECO-optCNN	PSO-optCNN	AVOA-optCNN
Accuracy	0.7508	0.7924	0.9739	0.9742	0.9682	0.9700	0.9795
AUC	0.8124	0.8593	0.9879	0.9821	0.9792	0.9796	0.9891
F1-score	0.4773	0.5226	0.8549	0.8571	0.8302	0.8247	0.8861
Precision	0.3608	0.4072	0.8862	0.8656	0.7774	0.9479	0.8799
Recall	0.7052	0.7294	0.8258	0.8488	0.8907	0.7299	0.8925

The proposed AVOA-optCNN method achieves an accuracy of 95.39% for the HMR 195 dataset and 97.95% for the GENSCAN training set, which is superior to its counterparts. The results further reveal that the Precision and Recall of the AVOA-based optCNN-HMR and optCNN-GenTrain models achieve the highest values compared to the existing and other optimization methods except for the precision value of the proposed method on the GENSCAN training set. In the GENSCAN training set, the PSO-optCNN precision value is higher than the AVOA-optCNN precision. However, the overall F1-score of the proposed method is higher than other methods. A good F1-score indicates that the model obtained low false negatives and low false positives by performing the weighted average of precision and recall values. The highest F1-score represents the better classification performance of the model. Furthermore, the AUC value of the AVOA-optCNN for the HMR195 dataset is 98.01% and for the GENSCAN training set is 98.91%, which is higher than other methods.

**Fig. 8:**
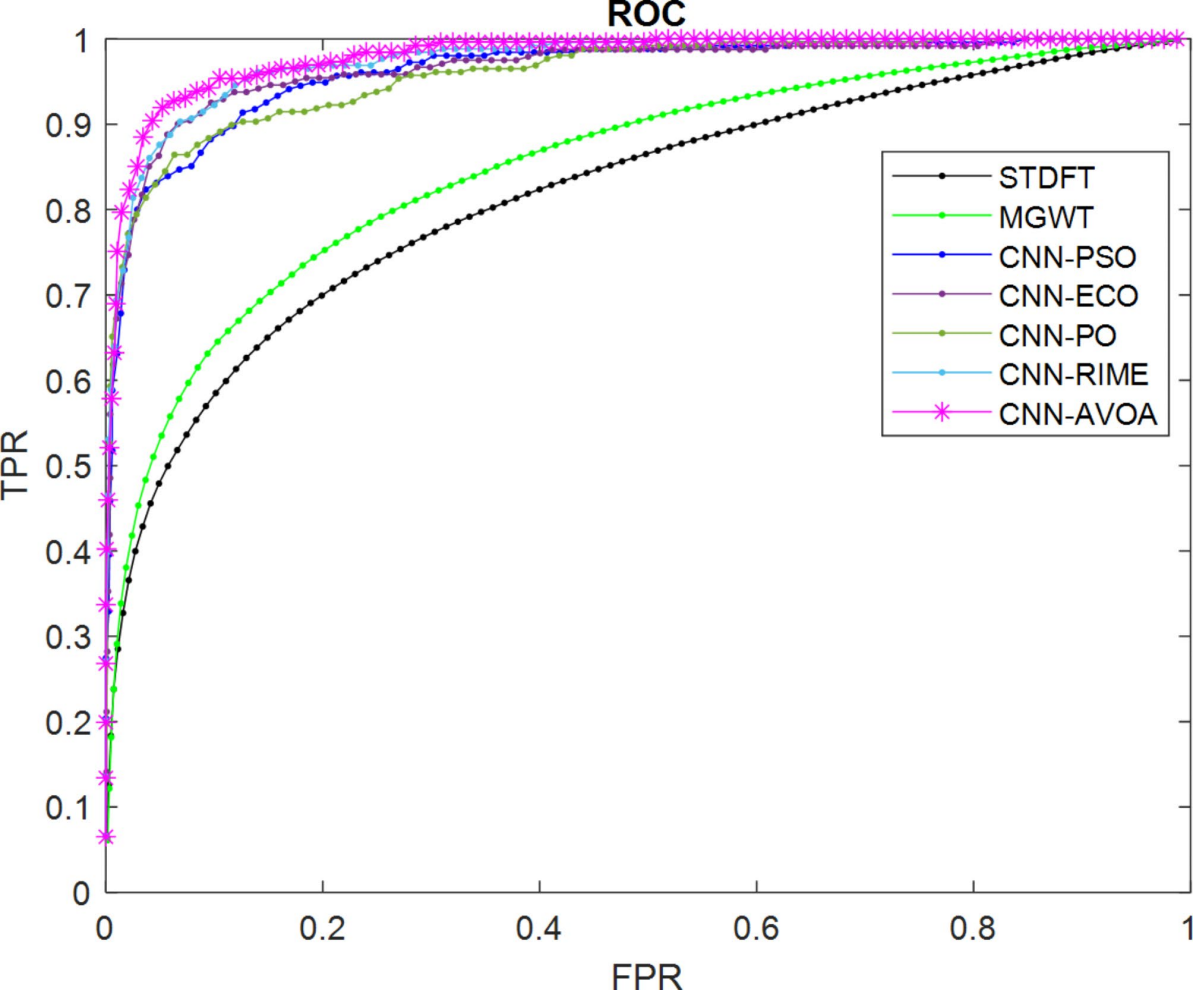
ROC curves for evaluating the performance of the proposed optCNN-HMR model.

**Fig. 9:**
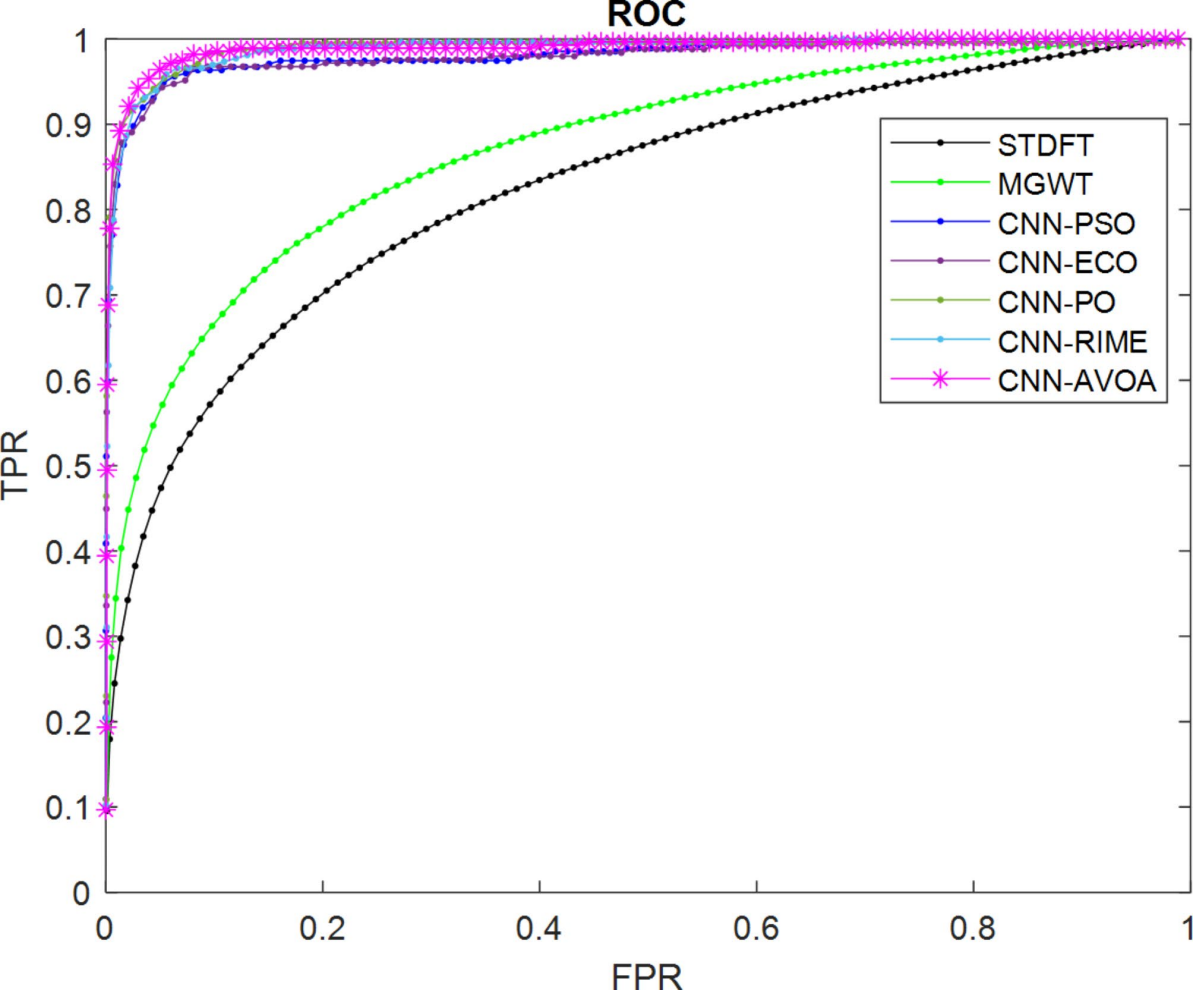
ROC curves for evaluating the performance of the proposed optCNN-GenTrain model.

Figures 8 and 9 depict the ROC curves of the proposed and other considered methods for the HMR195 dataset and the GENSCAN training set, respectively.

Similarly, for the dataset GENSCAN training set, the ROC curve for the proposed AVOA-optCNN and other methods are illustrated in Fig. 9.

A better AUC is achieved by the proposed model based on the efficient optimization properties of AVOA. The design of the AVOA algorithm focuses on balancing exploration and exploitation based on the search process, leading to enhancing the convergence speed and improving the accuracy in optimization problems. This expertise allows the model to learn more significant features during the training process and improves the classification performance.

**Fig. 10:**
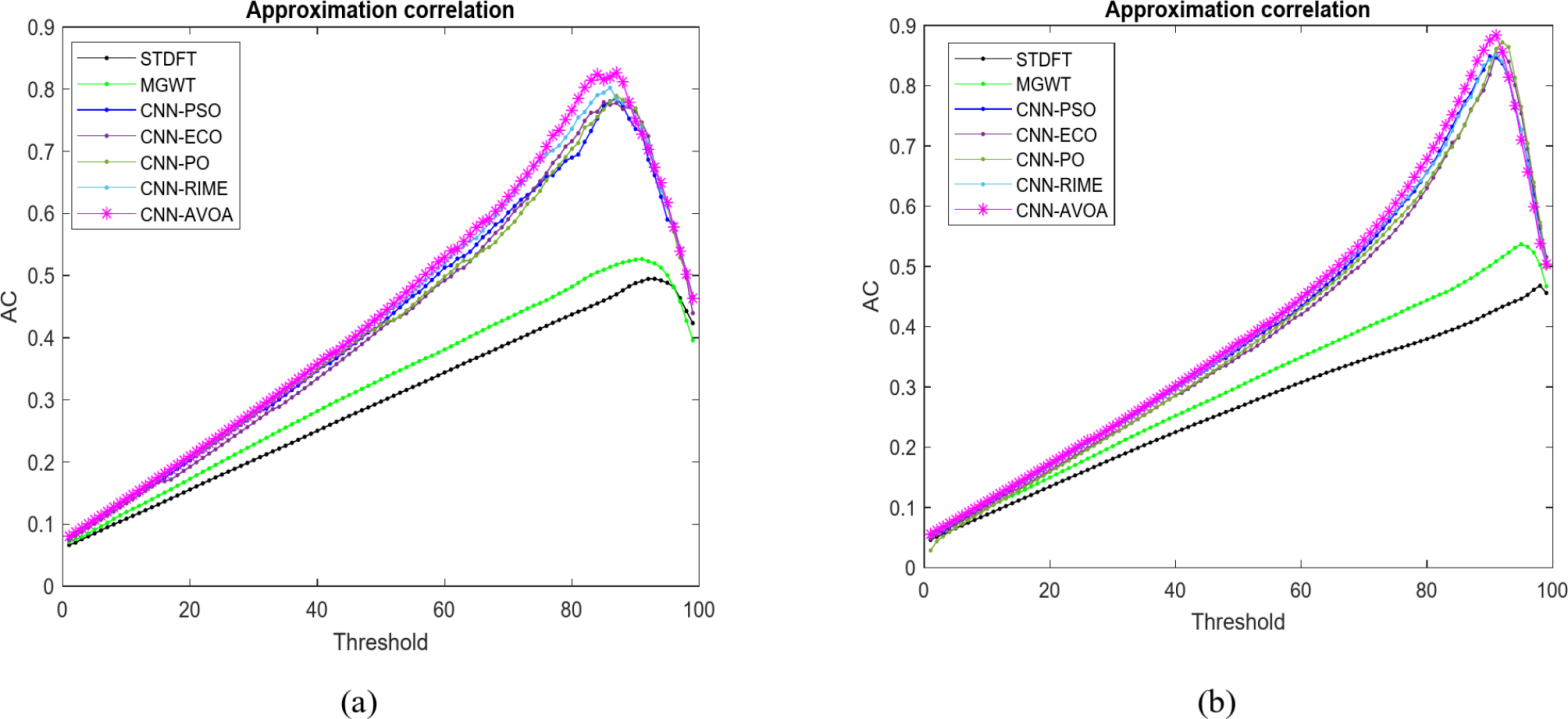
Approximation correlation (vs.) Threshold for evaluating the performance of the proposed (**a**) AVOA-optCNN-HMR model and (**b**) AVOA-optCNN-GenTrain model.

**Fig. 11:**
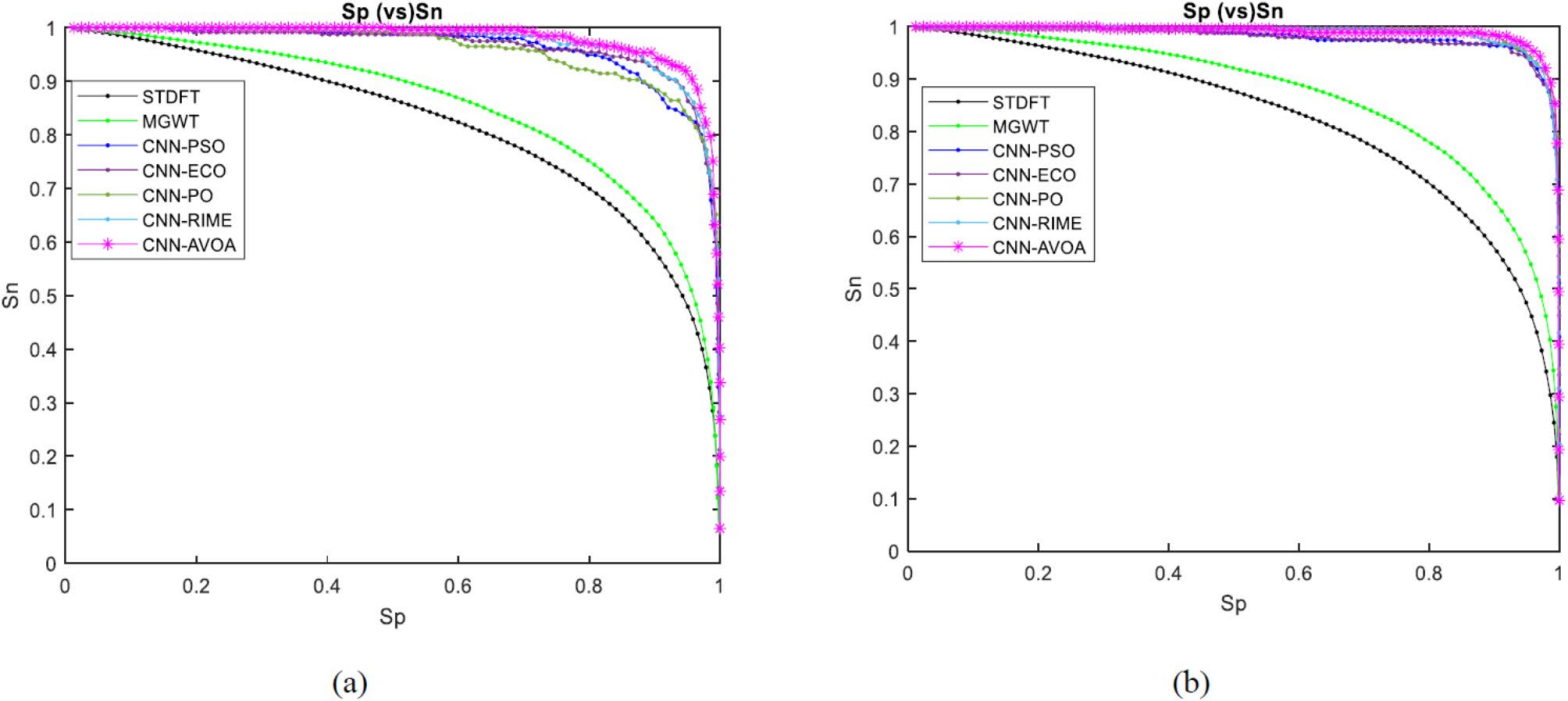
Sensitivity (vs.) Specificity for evaluating the performance of the proposed (**a**) AVOA-optCNN-HMR model and (**b**) AVOA-optCNN-GenTrain model.

**Fig. 12:**
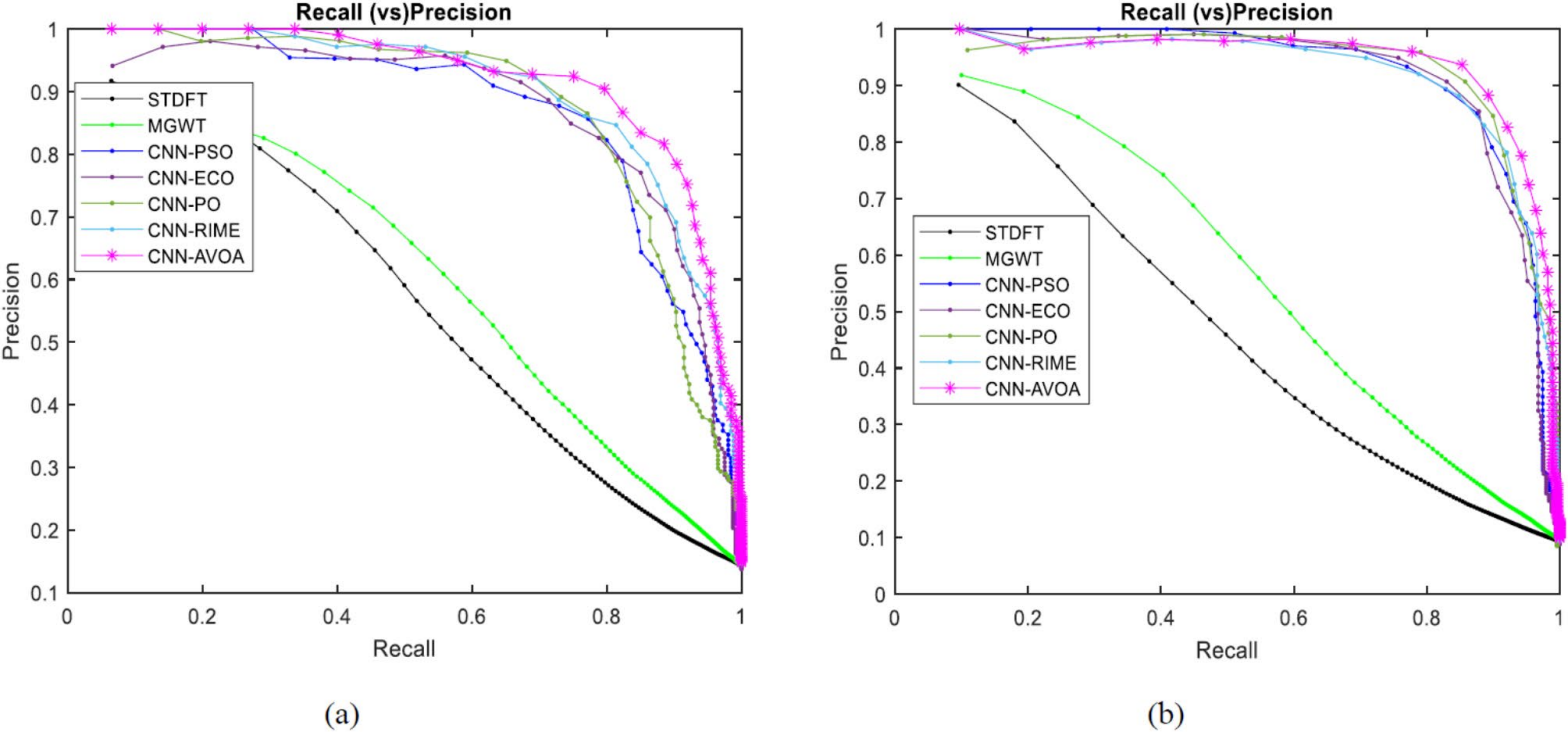
Precision (vs.) Recall for evaluating the performance of the proposed (**a**) AVOA-optCNN-HMR model and (**b**) AVOA-optCNN-GenTrain model.

**Fig. 13:**
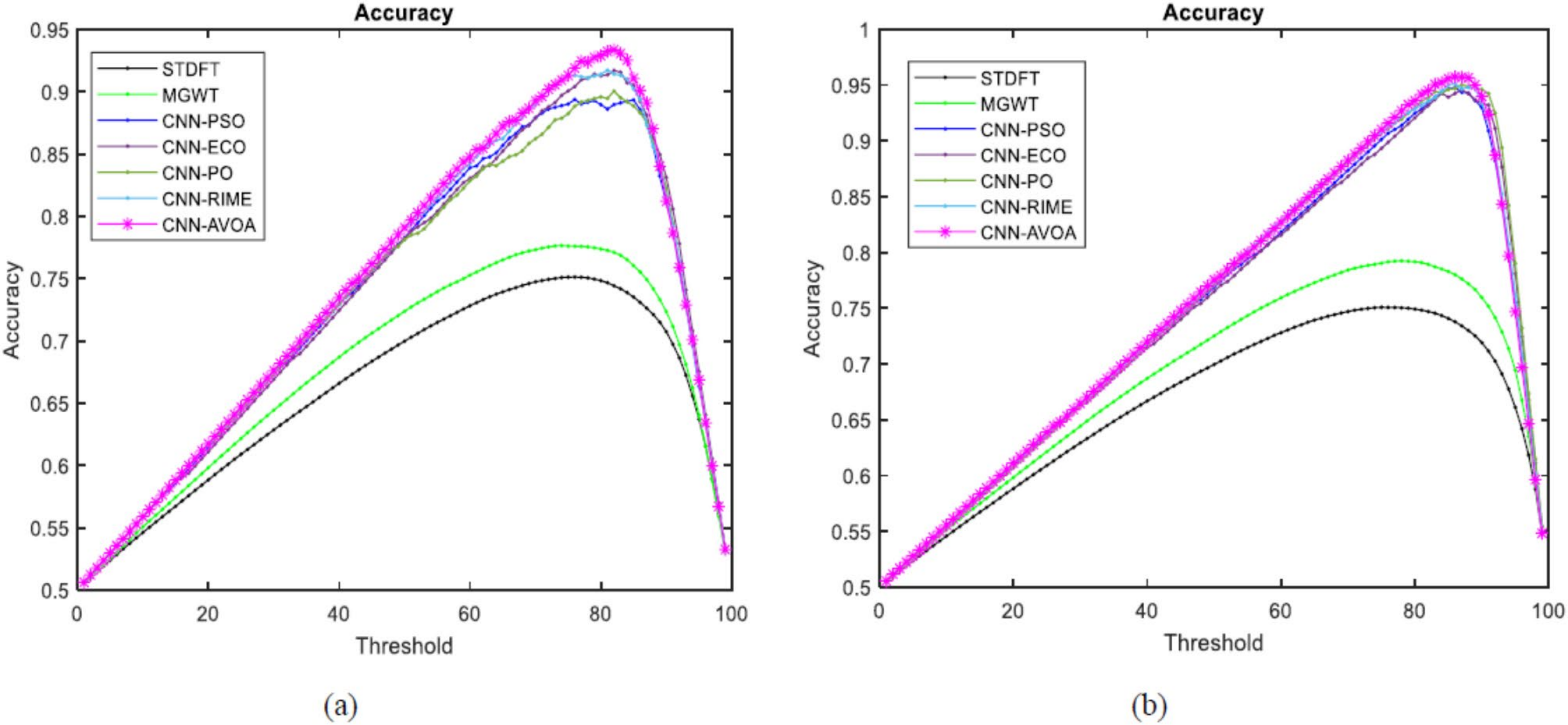
Accuracy (vs.) threshold for evaluating the performance of the proposed (**a**) AVOA-optCNN-HMR model and (**b**) AVOA-optCNN-GenTrain model.

We can examine how well the suggested model and other approaches perform at each of the potential threshold values by using the different graphs. Figures 10, 11, 12 and 13 represent the Approximation correlation (vs.) Threshold, sensitivity (vs.) specificity, Precision (vs.) Recall, and Accuracy(vs.) Threshold, respectively for the optCNN-HMR and optCNN-GenTrain models.

These graphs demonstrate that AVOA-optCNN performs significantly better than other optimization methods and existing methods for identifying the exons in eukaryotic DNA sequences.

## Conclusion

The proposed AVOA-based optCNN structure appears to be an effective hybrid model by automatically searching for a layered architecture of the CNN model with its associated hyperparameters optimized to achieve superior performance in the exons and introns classification task. The proposed approach is a simple-to-use, efficient, and powerful technique that may be applied to many classification problems. The efficacy of the proposed model is verified by comparing it with PSO, ECO, PO, and RIME-based optimized CNN models using the HMR 195 dataset and the GENSCAN training set. Finally, the performance of the proposed model is evaluated in terms of Accuracy, AUC, F1-score, Precision, and Recall using benchmark datasets. The experimental results demonstrate that the proposed method achieves superior performance than other state-of-the-art methods.

## Data Availability

The datasets used and analyzed during the current study are available from the corresponding author upon reasonable request.
